# Olive Oil and Soybean Oil Based Intravenous Lipid Emulsions, Liver Biochemistry and Clinical Outcomes

**DOI:** 10.3390/nu10060658

**Published:** 2018-05-23

**Authors:** Fabio Araujo, Tanis R. Fenton, Sara Lukmanji, Maitreyi Raman

**Affiliations:** 1 Department of Nutrition Services, Alberta Health Services, 3500 26 Ave NE, Calgary, AB T1Y 6J4, Canada; fabio.araujo@albertahealthservices.ca (F.A.); tfenton@ucalgary.ca (T.R.F.); 2 Community Health Sciences, Institute of Public Health, G055 3330 Hospital Drive, Calgary, AB T2N 4N1, Canada; 3 Faculty of Medicine, University of Calgary, G055 3330 Hospital Drive, Calgary, AB T2N 4N1, Canada; sara.lukmanji@ucalgary.ca

**Keywords:** IV lipid emulsion, olive oil, parenteral nutrition, soybean oil, Intralipid, Clinoleic

## Abstract

Intravenous lipid emulsions are an essential component of parenteral nutrition (PN). Omega-6 reducing strategies may improve outcomes, including reduced PN associated liver disease, however evidence to support this recommendation is insufficient. The primary objective was to compare serum alkaline phosphatase (ALP), among patients provided with either soybean oil (Intralipid) or predominantly olive oil (Clinoleic) lipid emulsions. In this quasi-experimental study, we reviewed the medical records of surgical and medical adult patients who received lipid emulsions for at least seven consecutive days. Among the 206 patients (110—Intralipid, 96—Clinoleic) there was no significant difference in ALP and remaining liver function tests within 2 weeks of PN therapy initiation between groups, even after control for lipid doses. Macronutrient dosing was similar. Triglyceride level was higher by 0.7 mmol/L in the Clinoleic group; confidence interval 0.21 to 1.1; *p* = 0.004. The 30-day mortality, length of hospital stay, and proportion of patients admitted to intensive care were not significantly different. The Clinoleic group had a higher infection rate (36% vs. 22%, *p* = 0.031) and longer intensive care stays (*p* = 0.045). Well-designed randomized clinical trials comparing these lipid emulsions are necessary to confirm Intralipid superiority over Clinoleic in relation to infections and serum triglycerides.

## 1. Introduction

Intravenous lipid emulsions (IVLE) are essential components of parenteral nutrition (PN) solutions, providing energy and essential fatty acids. Soybean oil based IVLE Intralipid (20% Intralipid, Fresenius Kabi, Uppsala, Sweden) has been the staple IVLE in North America for PN therapy [1,2]. It is rich in the polyunsaturated fatty acid linoleic acid (n-6 PUFA) (52%), which functions as a precursor of arachidonic acid, giving rise to eicosanoids that may predispose a more pro-inflammatory response, potentially adversely affecting immunologic cell functions [3,4].

PN-induced cholestasis is a description of the onset of liver disease in the context of administration of intravenous nutrition in patients with temporary or permanent intestinal failure. Other terms commonly used to describe the condition are PN-associated liver disease (PNALD), PN-associated cholestasis (PNAC), and intestinal failure-associated liver disease (IFALD) [5]. All three terms are often used interchangeably. Excess calories, high doses of n-6 PUFA, high dextrose doses and the phytosterol content of IVLE have been associated with PNALD in the adult population [6,7,8,9,10,11,12,13].

The pathogenesis of PNALD is complex and multifactorial, and associated with a decrease in bile flow, resulting in cholelithiasis and bacterial overgrowth leading to gut translocation of endotoxins into the portal circulation [14,15]. PNALD can present as mild liver enzymes elevation with resolution following discontinuing PN, in addition to cholestasis, steatosis and steatohepatitis [11]. Liver enzyme elevation can be observed as early as five days after PN start and may peak after 2–4 weeks [16,17,18].

Clinoleic (20% Clinoleic, Baxter Corporation, Mississauga, ON, Canada; oil composition: 80% olive oil, 20% soybean oil) is a novel IVLE rich in monounsaturated acid oleic acid (n-9 MUFA). Its effect on the immune system is incompletely understood. In vitro studies have shown that MUFA has a neutral effect on inflammation [18,19,20]. An n-9 MUFA rich emulsion could be less prone to lipid peroxidation since it has a single unsaturation and a higher content of antioxidant alpha-tocopherol (Table 1) [21].

Clinoleic may be a superior choice to Intralipid, however the existing human trial evidence is not clear. Most of the human randomized trials of Clinoleic emulsion have been conducted on preterm infants [22] and home PN patients [23]. There is only limited information available to inform the decision making regarding lipid choices between Intralipid and Clinoleic among adult surgical and medical patients who require PN in the short term. A randomized clinical trial of Clinoleic versus Intralipid found no difference in the outcomes assessed (glycemia, length of stay, nosocomial infection, acute renal failure, inflammation, or oxidative stress markers), but liver function tests were not reported [24]. While a few small studies have shown that a predominantly olive oil based IVLE is well tolerated and safe [24,25], the clinical relevance of this IVLE remains unclear based on observations in small randomized clinical trials.

Since limited data is available for the adult population reporting on the experience with olive oil based IVLE, we designed a quasi-experimental study to review our experience with Clinoleic compared to Intralipid. Given biological plausibility for lower risk of PNALD with Clinoleic use, we hypothesized that biliary tree serum enzymes would be lower in this group on the follow up blood work when compared to the Intralipid group. We used serum alkaline phosphatase (ALP) as a surrogate marker of cholestasis as this test is part of the standard pre-PN blood work in our hospitals. Our primary objective was to compare the impact of Intralipid and Clinoleic IVLE on serum ALP—pre-PN to after one week of PN (i.e., between day 8 to 16 post-PN initiation)—while controlling for the ordered lipid dosing and baseline levels. Secondary objectives were to assess if there were differences between the IVLEs on the remaining liver function tests, lipid dosing, incidence of infectious complications, length of stay in hospital, and 30-day mortality.

## 2. Materials and Methods

This retrospective quasi-experimental chart review was conducted in three tertiary care hospitals in Calgary, AB, between 1 July 2012 to 30 June 2013 and 1 July 2014 to 30 June 2015. Standard soybean oil-based therapy, Intralipid, was the only available IVLE in Calgary, AB until July 2013, at which time predominantly olive oil Clinoleic was approved as an alternative in the hospital formulary, accounting for the dates chosen for the study. Ethics approval from the Conjoint Health Research and Ethics Board at the University of Calgary was obtained prior to the initiation of the study.

### 2.1. Study Population

Study patients included adult patients (age > 18 years old) admitted to hospital who received PN with Intralipid or Clinoleic IVLEs for at least seven consecutive days. Study exclusion criteria included baseline liver disease, home PN prior to admission, ALP and total bilirubin (TB) not available within 3 days prior to PN start as well as between days 8 to 16 post PN start, receipt of Diprivan 1%^®^ (Propofol-AstraZeneca Canada Inc., Mississauga, ON, Canada) during PN support period, enteral nutrition providing greater than 600 Kcal daily for longer than half of time period on PN, and/or oral intake of greater than 50% of hospital meal tray contents for longer than half of the PN support time period. Enteral and oral intake data, documented by nursing staff, were collected from medical charts’ flow sheets.

### 2.2. Study Outcome Measures

The pre-declared primary outcome variable was the difference between groups’ serum ALP levels, 8 to 16 days after PN initiation of either Intralipid or Clinoleic IVLE. Pre-declared secondary outcome measures included comparing the (a) differences in serum levels of alanine aminotransferase (ALT), gamma-glutamyl transferase (GGT), TB, bilirubin direct (BD), and triglycerides (TG) measured between day 8 to 16; (b) dosing of IVLE prescriptions from days 3 to 16 (presuming that it takes 2 days to reach goal dose of PN and macronutrients); (c) all-cause mortality by 30 days; (d) length of hospital stay; and (e) infectious complications incidence during period on PN, defined as (i) positive sputum cultures plus characteristic chest X-ray findings of lobar consolidation or diffuse bilateral infiltrates requiring antibiotic therapy (pneumonia); (ii) catheter and blood revealing the same microorganism (catheter infection); (iii) positive peripheral blood cultures alone, and no other source identified (bacteremia); (iv) positive urine cultures requiring antibiotics (urinary tract infection) and/or (v) positive stool cultures. Analyses that were not pre-specified were not conducted. New infectious events were recorded from the second day of PN until the day after PN discontinuation. Each new infection was recorded once for each patient, even if there was more than one sample with positive microbiology culture. Catheter related infections were confirmed if there was the presence of the microorganism isolated from the catheter, concomitant with peripheral positive cultures. An infectious episode was considered non-catheter related if another source was confirmed positive with same microorganism identified in the peripheral culture only.

### 2.3. Statistical Analyses

Descriptive statistics, specifically proportions or means with standard deviations, were used to describe participants’ demographic characteristics, anthropometrics, and relevant medical history (i.e.**,** diagnosis on admission, surgical history and indication for PN). Differences between the Intralipid and Clinoleic groups were analyzed using the *t*-test for continuous variables and Fisher’s Exact Test for the categorical variables. Multivariable linear regression analyses were undertaken to test for differences in the change from baseline laboratory values between lipid groups. A priori we planned to control for variables that differed between the groups with *p*-values < 0.10 and baseline levels. Lipid doses ordered during the period on PN differed between the groups (*p* = 0.018), so were also controlled for.

Based on our sample size calculation that assumed an alpha of 0.05, power of 80%, and a standard deviation of 15 units [24], we estimated the need for 120 patients in each group to identify a difference in ALP of 30 units/L. Further, using the aforementioned alpha and power, and assuming a standard deviation of 0.2 g/kg/day [26], we estimated 64 patients in each group were required to determine a difference in lipid ordering practices.

## 3. Results

**Figure 1:**
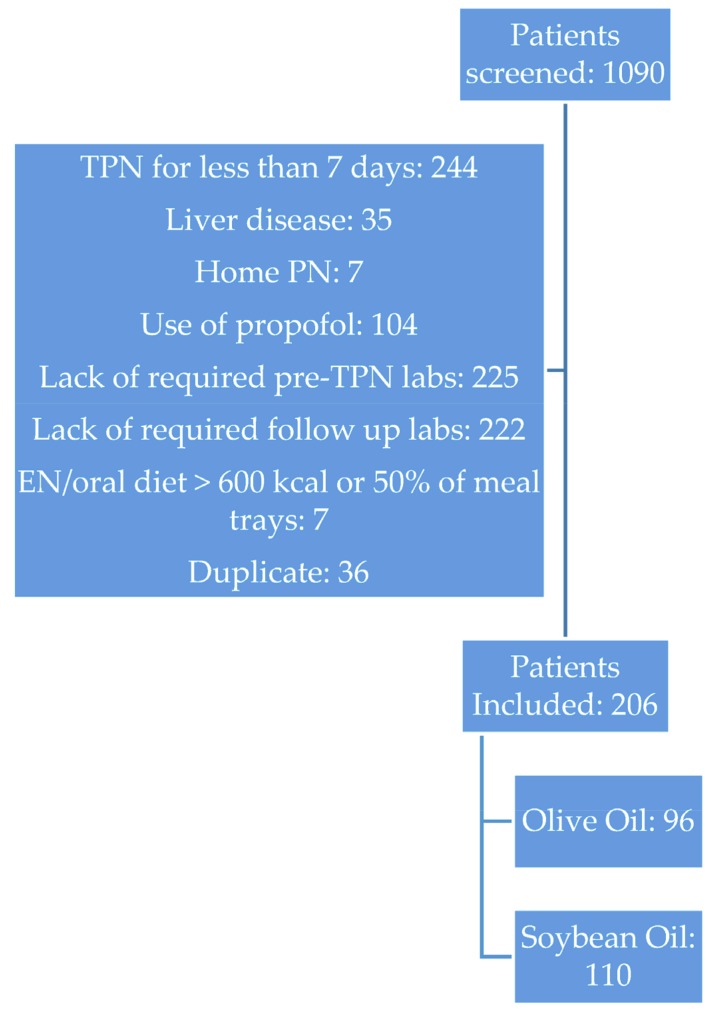
Screening, inclusion and exclusion criteria.

Of the 1090 patient charts which were reviewed, 206 patients (110 in the Intralipid and 96 in the Clinoleic groups) met the inclusion criteria (Figure 1). The main reasons for non-inclusion in the study were lack of serum biochemistry values at the time of follow up (51%) and total PN for less than 7 days (28%) (Figure 1). Baseline patient characteristics between both groups of patients were similar (Table 2). Gastrointestinal illness or surgery were the primary indications for PN. There were no significant differences observed between the two groups for age, sex, BMI, nutrition status, diagnoses, or PN indications.

### 3.1. Primary Outcome

There was no significant difference in ALP levels between the IVLE groups after 2 weeks of PN (Table 3). In the multivariable model controlled for the dosing of lipid and baseline levels, the change in ALP from baseline was not associated with IVLE type (Table 3) or dosing of lipid.

#### Parenteral Nutrition Dosing

Total PN energy and amino acid delivered in both groups were similar. Lipid dosing was statistically significantly higher (0.83 vs. 0.78 g/Kg) and dextrose dosing was significantly lower (3.7 vs. 4.0 g/Kg) in patients receiving Clinoleic compared to Intralipid (Table 2).

### 3.2. Secondary Outcomes

Secondary outcomes are detailed in Table 3 and Table 4. In the simple analysis, follow up levels of the remaining laboratory markers (ALT, GGT, TB, and BD) were not significantly different between the groups receiving the two types of lipid, except for TG, which were higher in the patients that received Clinoleic compared to the group that received Intralipid by 0.67 mmol/L, confidence interval 0.21 to 1.1 (*p* = 0.004, Table 3). In the multivariable models controlled for ordered lipid doses and baseline levels, lipid type was associated with change of serum TG, which were higher in the Clinoleic group (*p* = 0.002, Table 3), but none of the other lab markers. The number of observations was low for changes in direct bilirubin and triglycerides (*n* = 14 and 75, respectively). Lipid dose was not statistically predictive of liver enzymes, bilirubin, or TG levels in the multivariable models (data not shown).

While the analyses—both the univariate and controlled for baseline levels and lipid doses—were not significantly different between the two lipids except for TG, there were differences between the univariate and controlled estimated differences (Table 3), which indicates that either or both of the controlled variables (baseline and ordered doses) influentially confounded the univariate uncontrolled analyses to some degree.

The incidence of all infectious complications were significantly lower in patients receiving Intralipid (22%) compared with Clinoleic (36%, *p* = 0.031). The infections by type did not differ between the groups. The 30-day mortality and proportion of patients admitted to the ICU did not differ significantly between the groups (Table 4). The length of hospital stay did not differ between the groups but the length of ICU stay was shorter for the Intralipid group compared to the Clinoleic group (Table 4, *p* = 0.045).

## 4. Discussion

Our short term quasi-experimental study revealed some differences: higher serum TG, higher rate of infections, and longer length of ICU stay for patients that received Clinoleic compared to those who received Intralipid IVLE. The higher serum TG level remained significantly higher after controlling for lipid dose and baseline TG levels. Liver biochemistry (ALP, ALT) and liver function measured by TB and BD were similar between medical and surgical acute inpatients on PN receiving Intralipid or Clinoleic IVLE 8–16 days after starting PN, even after controlling for lipid doses and baseline levels. Our Intralipid and Clinoleic groups were similar in terms of age, gender, weight status, BMI, type of diagnosis, type of indication for PN start, and whether they received clinically similar lipid dosing (0.78 g/Kg in the Intralipid group vs. 0.83 g/Kg in Clinoleic group).

The prescribed lipid doses in our study were conservative, potentially explaining the lack of difference between the groups. The conservative lipid prescribing practices infer caution exercised by the treating dietitian with both lipids, despite previous reports that doses of up to 1.2 g/Kg are safe and well tolerated [27] and despite the expected biologic rationale for superiority of olive oil compared with soybean oil based IVLE [26,28,29,30]. Our findings are consistent with findings by previous studies. A similar difference in infection rates (57% vs. 43%, difference = 14%) to our study (36% vs. 22%, difference = 14%) was seen in a randomized clinical trial of Clinoleic or Intralipid in medical-surgical patients [24]. A subgroup analysis from a systematic review of critical care patients revealed non-significantly higher rates of infection among patients receiving olive oil based IVLE compared to long-and-medium-chain-triglyceride mixtures (RR = 1.23, 95% CI 0.92, 1.63, *p* = 0.16, heterogeneity I^2^ = 0%) [31]. Conversely, in a randomized controlled study with surgical adult patients (which differed by the PN delivery system (3-chamber system vs. lipids separate) and electrolyte dosing (standard dosing vs. custom prescription) as well as differing in IVLE) [25], patients receiving olive oil based IVLE experienced significantly fewer overall infections and higher albumin and pre-albumin compared with patients receiving Intralipid. In a separate non-randomized trial, no differences in catheter infections were observed along with ALT and TB between patients matched on age and gender after seven days of PN with either soybean or olive oil based IVLE post abdominal surgery, however ALP was significantly higher in the olive oil-based emulsion group [32].

Whether the higher observed infection rates in the Clinoleic group in our study are due to the lipids or confounding by group differences is not readily apparent. The baseline characteristics for patients in both groups were similar, and likely not contributory to this finding. The differences in infection rates between the groups were not due to the duration of PN, which was not statistically or clinically different between the two. The mean dextrose dosing was lower in the olive oil based IVLE group, and thus could not be implicated in contributing toward the higher infectious rates. Lipid doses were clinically similar. Umpierrez and colleagues [24] demonstrated that in a cohort of adult ICU patients, there were no differences in systemic inflammatory markers, oxidative stress, or immune markers between Clinoleic and Intralipid. These similar pathophysiologic mechanisms could explain the lack of superiority of Clinoleic over Intralipid regarding infectious outcomes, but does not explain the increased infectious rates observed in this study. Larger well-designed randomized trials are needed to determine whether this finding is due to the different lipid infusions or due to chance or confounding by differences between our groups.

There are a few limitations in our study. This is a retrospective observational study where the subjects in different time periods were not randomized to the two lipid types. The differences in infections, length of ICU stay, and TG levels may be due to other factors that may have differed between the groups and time periods, such as disease etiology, disease severity, and comorbidities. We did not capture data on blood sugars, nor did we identify the proportion of patients with diabetes within each group. Poorly controlled blood sugars can have a substantial impact on the rates of infection. Despite similar baseline demographic and PN characteristics, individual patients and clinical course may be different, potentially explaining some study findings. Conceivably, the longer ICU and hospitalization of the cohort that received Clinoleic may reflect a sicker patient population.

## 5. Conclusions

Our study did not show statistically significant differences between Intralipid and Clinoleic IVLE in terms of liver enzymes, liver function tests, and length of stay in hospital or mortality. We observed a higher infection incidence in the Clinoleic group, however that did not lead to higher mortality rate. Serum TG and ICU length of stay were higher in the patients on Clinoleic compared to Intralipid. These findings should be considered hypothesis generating, rather than definitive results as they were from an observational study, and well-designed large randomized controlled trials are needed to better elucidate the impact of frequently used IVLEs on patient outcomes.

## Figures and Tables

**Table 1:** Composition of lipid emulsions used in the study.

	Soybean Oil Lipid Emulsion (Intralipid 20%)	Olive Oil Lipid Emulsion (Clinoleic 20%)
1000 mL contains:		
Soybean oil	200 g	40 g
Olive Oil	0	160 g
Glycerol Anhydrous	22 g	22.5 g
Egg phospholipids	12 g	12 g
Water for injection q.s.ad	1000 mL	1000 mL
Sodium Oleate	-	0.3 g
Alpha-tocopherol	9–14 mg	25–35 mg
Phytosterols	348 ± 33 mg	327 ± 8 mg
Fatty acid profile (%)		
Saturated	15%	15%
Mono-unsaturated	24%	65%
Poly-unsaturated	61%	20%
Oleic Acid	22%	62%
Linoleic Acid	52%	18%
∞-Linolenic Acid	8%	2%
Linoleic: ∞-Linolenic	7:1	9:1

Clinoleic 20%: Product Monograph; Baxter Corporation: Deerfield, IL, USA, 2014. Intralipid 20%, Product Monograph; Fresenius Kabi AB: Hongkong, China, 2013.

**Table 2:** Characteristics of patients.

	Soybean Oil Based Intralipid^®^	Olive Oil Based Clinoleic^®^	*p*-Value
Number of patients	110	96	
Mean age (years) *	63.6 ± 14.3	63.6 ± 15.8	0.93
Male	48% (53/110)	50% (48/96)	0.89
Mean weight (Kg)	71.7 ± 20.2	75.2 ± 26	0.24
Mean BMI (Kg/m^2^) *	27.3 ± 20.7	26.8 ± 7.9	0.84
Nutrition Status (%/patients)			0.15
Well Nourished	4% (4)	4% (4)	
Mild or moderate malnutrition	14% (15)	15% (14)	
Severe malnutrition	19% (21)	7% (7)	
Not reported	63% (70)	74% (71)	
Diagnosis on admission			0.66
GI related conditions—Surgical	44% (48/110)	39% (37/96)	
GI related conditions—Non Surgical	8% (9/110)	13% (12/96)	
Cancer	33% (36/110)	36% (35/96)	
Pancreatitis	5% (6/110)	5% (5/96)	
Cachexia/Malnutrition	2% (2/110)	3% (3/96)	
Other	8% (9/110)	4% (4/96)	
Total surgical patients (GI + non GI)	74% (81/110)	79% (76/96)	
Indication for TPN			0.68
GI related Conditions	53% (58/110)	62% (59/96)	
Unsuccessful PO or EN	20% (22/110)	12% (11/96)	
Ileus	8% (9/110)	7% (7/96)	
Pancreatitis	4.5% (5/110)	5% (5/96)	
Pre-op Boost	4.5% (5/110)	4% (4/96)	
Non-specified	10% (11/110)	10% (10/96)	
Parenteral Nutrition days 3–16			
Energy (Kcal/Kg)	27.7 ± 4.4	27.4 ± 4.3	0.54
Lipid dosing (g/Kg)	0.78 ± 0.11	0.83 ± 0.16	0.018
Amino Acid dosing (g/Kg)	1.5 ± 0.3	1.6 ± 0.3	0.07
Dextrose dosing (g/Kg)	4.0 ± 0.9	3.7 ± 0.82	0.010

* Plus-minus values are means ± standard deviation.

**Table 3:** Liver biochemistry, bilirubin, and serum triglycerides by lipid type, Intralipid^®^ versus Clinoleic^®^, after 2 weeks of parenteral nutrition *.

		Univariable Analysis					Analysis Controlled for Lipid Dose and Baseline Levels			
Laboratory	*n*	Intralipid^®^	Clinoleic^®^	Average Difference	Confidence Interval	*p*-Value	*n*	Average Difference	Confidence Interval	*p*-Value
Alkaline phosphatase (U/L)	110, 96	160 ± 133	175 ± 178	14.5	−28.4 to 57.5	0.51	206	23.0	−7.3 to 53.4	0.14
Gamma-glutamyl transferase (U/L)	46, 48	185 ± 379	144 ± 211	−41.0	−167 to 85.2	0.52	116	46.3	−6.7 to 99	0.09
Alanine aminotransferase, (U/L)	55, 53	36 ± 34	54 ± 72	18.3	−3.1 to 39.8	0.09	204	54.4	−5.0 to 114	0.07
Bilirubin total, (umol/L)	110, 96	14 ± 26	14 ± 26	0.2	−7.0 to 7.3	0.97	206	−2.8	−9.1 to 3.6	0.40
Bilirubin direct, (umol/L)	24, 17	18 ± 25	23 ± 9.3	5.8	−14 to 26	0.56	16	−11.1	−30 to 8.1	0.23
Triglycerides, (mmol/L)	64, 57	1.3 ± 0.7	2.0 ±1.7	0.67	0.21 to 1.1	0.004	75	0.74	0.28 to 1.20	0.002

* Plus-minus values are means ± standard deviation. Average differences and confidence intervals represent the difference between the means for Clinoleic^®^ minus Intralipid^®^.

**Table 4:** Other outcomes *.

	Soybean Oil Based Intralipid^®^	Olive Oil Based Clinoleic^®^	*p*-Value
Total period on TPN (days)	25 ± 37	21 ± 24	0.36
Median (IQR)	16 (12–25)	14 (11–23)	
Patients admitted to ICU	25% (28/110)	32% (31/96)	0.28
Length of Stay in ICU (days)	6 ± 7	12 ± 14	0.045
Length of stay in hospital (days)	43 ± 49	54 ± 63	0.15
30 day mortality	11% (12/110)	14% (14/96)	0.53
Incidence of infections	22% (24/110)	36% (35/96)	0.031
Infection complications:	40.7% (24/59)	59.3% (35/59)	0.23
Non-catheter related bacteremia	5	9	
Catheter related Sepsis	2	5	
Urine (UTI)	15	19	
Positive Sputum + Radiographic Evidence of Pneumonia (Lung infections)	1	6	
Stool (C. diff)	3	1	

* Plus-minus values are means ± standard deviation.
